# Mesenchymal stem cells overexpressing Sirt1 inhibit prostate cancer growth by recruiting natural killer cells and macrophages

**DOI:** 10.18632/oncotarget.12737

**Published:** 2016-10-18

**Authors:** Yang Yu, Qingyun Zhang, Qinggui Meng, Chen Zong, Lei Liang, Xue Yang, Rui Lin, Yan Liu, Yang Zhou, Hongxiang Zhang, Xiaojuan Hou, Zhipeng Han, Jiwen Cheng

**Affiliations:** ^1^ Department of Urology, Affiliated Tumor Hospital of Guangxi Medical University, Nanning, People's Republic of China; ^2^ Tumor Immunology and Gene Therapy Center, Eastern Hepatobiliary Surgery Hospital, The Second Military Medical University, Shanghai, People's Republic of China; ^3^ The Fifth Department of Chemotherapy, Affiliated Tumor Hospital of Guangxi Medical University, Nanning, People's Republic of China

**Keywords:** prostate cancer, mesenchymal stem cells, Sirt1, nature killer cells, C-X-C motif chemokine ligand 10, Immunology and Microbiology Section, Immune response, Immunity

## Abstract

Prostate cancer (PCa) has become the second leading cause of male cancer-related mortality in the United States. Mesenchymal stem cells (MSCs) are able to migrate to tumor tissues, and are thus considered to be novel antitumor carriers. However, due to their immunosuppressive nature, the application of MSCs in PCa therapy remains limited. In this study, we investigated the effect of MSCs overexpressing an NAD-dependent deacetylase sirtuin 1 (MSCs-Sirt1) on prostate tumor growth, and we analyzed the underlying mechanisms. Our results show that MSCs accelerate prostate tumor growth, whereas MSCs-Sirt1 significantly suppresses tumor growth. Natural killer (NK) cells and macrophages are the prominent antitumor effectors of the MSCs-Sirt1-induced antitumor activity. IFN-γ and C-X-C motif chemokine ligand 10 (CXCL10) are highly expressed in MSCs-Sirt1 mice. The antitumor effect of MSCs-Sirt1 is weakened when CXCL10 and IFN-γ are inhibited. These results show that MSCs-Sirt1 can effectively inhibit prostate cancer growthrecruiting NK cells and macrophages in a tumor inflammatory microenvironment.

## INTRODUCTION

Prostate cancer (PCa) is the most common cancer in men, and the second leading cause of male cancer-associated mortality [1, 2]. Early staged and localized PCa can be well controlled by prostatectomy or radiotherapy. For advanced PCa, androgen deprivation therapy (ADT) is currently considered as the most effective treatment, giving a 70% initial effective rate [3]. However, there are still many PCa patients who are not responsive to ADT. Furthermore, most androgen-responsive PCa patients develop androgen-resistant PCa after ADT. Thus, it is essential to find new effective strategies for PCa treatment.

Mesenchymal stem cells (MSCs) are a heterogeneous subset of stromal stem cells, which represent multipotent cells capable of differentiation into various lineages, including adipose, osteogenic, and chondrogenic tissues [4]. Based on their low immunogenicity, MSCs are believed to be a promising stem cell population for clinical applications [5]. Recently, MSCs gained attraction for their immunomodulatory effect in inflammatory microenvironment [6, 7]. MSCs can migrate to inflamed tissues where they can inhibit the release of pro-inflammatory cytokines, thus inducing peripheral tolerance and damaged cells survival [8]. MSCs are also an important component of tumor inflammatory microenvironment, assisting tumor escape from immunosurveillance and contributing to tumor development [9–12]. However, the therapeutic potential of MSCs in cancer treatment needs to be investigated to expand their applications.

Sirtuin 1 (Sirt 1) is an NAD-dependent deacetylase that is the closest mammalian homologue of the yeast Sir2, which regulates yeast lifespan [13]. In mammalian cells, Sirt1 regulates several biological functions, such as aging, metabolism, DNA damage, and tumor development [14]. Sirt1 is expressed in human MSCs, and overexpression of Sirt1 in aged MSCs reverses the senescence phenotype and stimulates cell proliferation [15]. However, the exact effect of MSCs overexpressing Sirt1 on tumor development remains unclear.

In this study, we constructed MSCs overexpressing Sirt1 (MSCs-Sirt1), and investigated the effect of MSCs-Sirt1 on PCa growth *in vivo*. We also analyzed the mechanisms underlying the regulatory effect of MSCs-Sirt1 on PCa tumor growth. Our results show that MSCs-Sirt1 inhibit PCa tumor growth, suggesting that they might represent a new potential strategy for PCa therapy.

## RESULTS

### MSCs-Sirt1 inhibit prostate tumor growth

**Figure 1:**
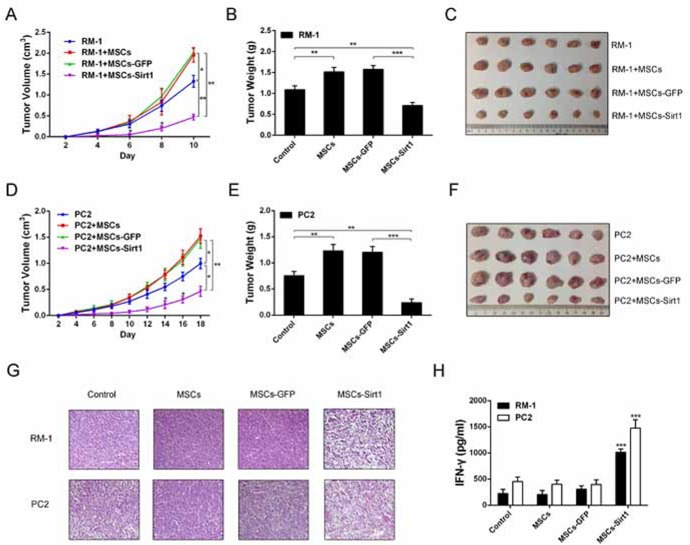
Effect of MSCs-Sirt1 on prostate cancer growth Tumor growth were detected per 2 days after subcutaneous administration with prostate cancer cells. The width and length of **A.** RM-1 and **D.** PC2 tumors were measured by calipers, then calculated the volume using the formula: volume=width^2^ × length × 0.5236. Tumor weights were measured after removed from mice on the **B.** 10^th^ or **E.** 18^th^ day. **C.** and **F.** Representive tumors were presented. **G.** H&E staining of tumor tissues was observed by microscope (original magnification: ×200). **H.** Blood samples were collected at the end of experiment, then serum inflammatory cytokines levels were determined by Bio-Plex Pro^TM^ mouse cytokine assay kit (Bio-Rad Laboratories, USA). Each group consists of 6 mice. *, *p* < 0.05; **, *p* < 0.01; ***, *p* < 0.001 compared with MSCs-GFP groups. MSCs-GFP, MSCs transfected GFP-Mock; MSCs-Sirt1, MSCs transfected GFP-Sirt1.

First, we constructed MSCs overexpressing Sirt1 (MSCs-Sirt1) by infecting MSCs with the adenoviral vector GFP-Sirt1 (Supplementary Figure 1). Then, we applied subcutaneously implanted tumor model in C57BL/6 mice to determine the effect of MSCs-Sirt1 on RM-1 and PC2 prostate cancer cells growth *in vivo*. As shown in Figure 1, RM-1 (Figure 1A) and PC2 (Figure 1D) cells co-injected with MSCs grew significantly faster than cells without MSCs, while RM-1 and PC2 cells co-injected with MSCs-Sirt1 exhibited the slowest growth. Compared with mice injected with RM-1 (Figure 1B) or PC2 (Figure 1E) cells alone, tumor weights were highest in mice co-injected with MSCs. However, when RM-1 or PC2 cells were co-injected with MSCs-Sirt1, tumor weights showed a significant reduction (Figure 1B and 1E). At the end of experiment, we proved the existence of MSCs-Sirt1 in tumor tissues (Supplementary Figure 2). These results indicate that MSCs-Sirt1 can significantly inhibit prostate cancer growth in mice.

### MSCs-Sirt1 induce IFN-γ expression *in vivo*

Several studies have reported that MSCs can enhance tumor development through immunosuppressive activity by inhibiting the release of pro-inflammatory cytokines [8, 12, 16]. In our study, histological analysis of tumors harvested from MSCs-Sirt1 treated mice demonstrated a dramatic change including low cell density and multinucleated cells with condensed chromatin staining and pyknosis, indicating necrosis (Figure 1G). Thus, we speculated that the antitumor effect of MSC-Sirt1 might correlate with the inflammatory responses *in vivo*. We analyzed the serum levels of cytokines IL-6, IL-8, IL-10, IFN-γ, and TNF-α in tumor-bearing mice. Interestingly, MSCs-Sirt1 treated mice had higher serum IFN-γ levels compared to mice in other groups (Figure 1H). There was no significant difference in other cytokines (Supplementary Figure 3A). These data indicate that MSCs-Sirt1 can induce IFN-γ production *in vivo*.

### MSCs-Sirt1 recruit NK cells to tumor tissues for tumor suppression

**Figure 2:**
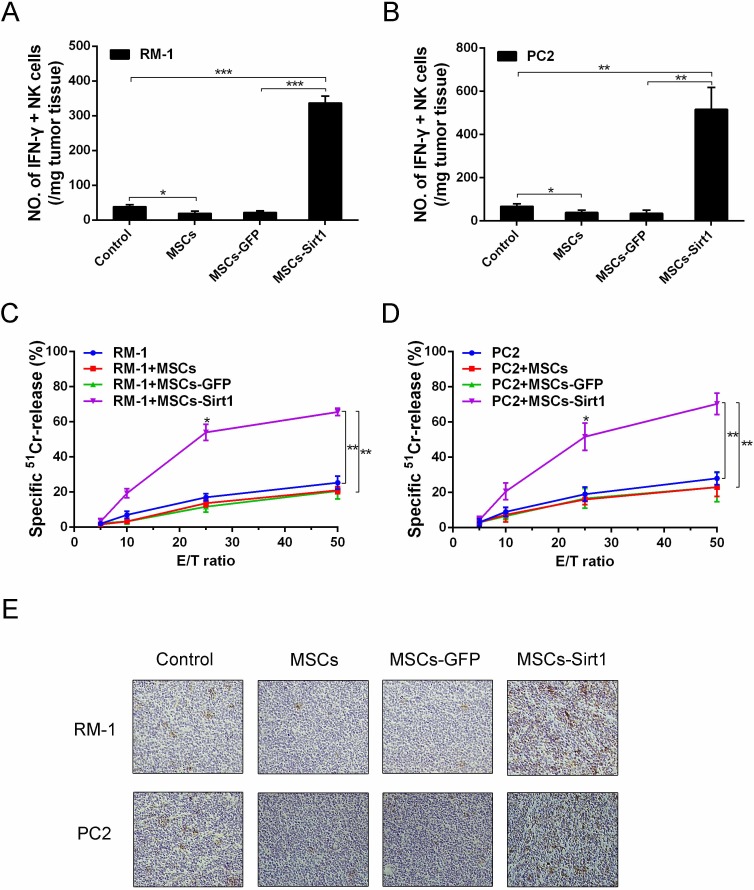
Tumor infiltrating NK cells evaluation in tumor-bearing mice Tumor infiltrating cells were isolated from **A.** RM-1 and **B.** PC2 tumor tissues, then analyzed by surface staining with anti-mouse NKp46 (eBioscience) for NK cells, followed by intracelluar IFN-γ staining. **C.** and **D.** Splenocytes (effector, E) were isolated from the mice and assayed against YAC-1 cells (target cell, T). Cell lysis was determined in triplicate and ^51^Cr isotope release assay at different effector-to-target cell (E/T) ratios were performed to determine the cytotoxic potential of effector populations. **E.** NK cells of tumor tissues were determined by immunohistochemistry assessment (original magnification: ×200). *, *P* < 0.05; **, *P* < 0.01; ***, *P* < 0.001.

Since IFN-γ is critically involved in the development of immune responses, we isolated and analyzed tumor-infiltrating lymphocyte cells by flow cytometry. As shown in Figure 2A and 2B, dramatically higher numbers of IFN-γ-secreting NK cells were detected in both RM-1 and PC2 tumor tissues of MSCs-Sirt1 groups compared to other groups. In contrast, there was no obvious difference in IFN-γ-secreting T and B cells (Supplementary Figure 3B and 3C). We also isolated splenocytes to detect the cytolytic NK activity. The data showed that there was a significant enhancement of cytolytic NK activity in the MSCs-Sirt1 group compared to other groups (Figure 2C and 2D). Moreover, we analyzed tumor expression of CD49b, a marker for NK cells. Compared with control group, the MSCs group showed a marked decrease in number of CD49b-positive cells. Conversely, there was an apparent increase of CD49b-positive cells in the MSCs-Sirt1 group (Figure 2E).

**Figure 3:**
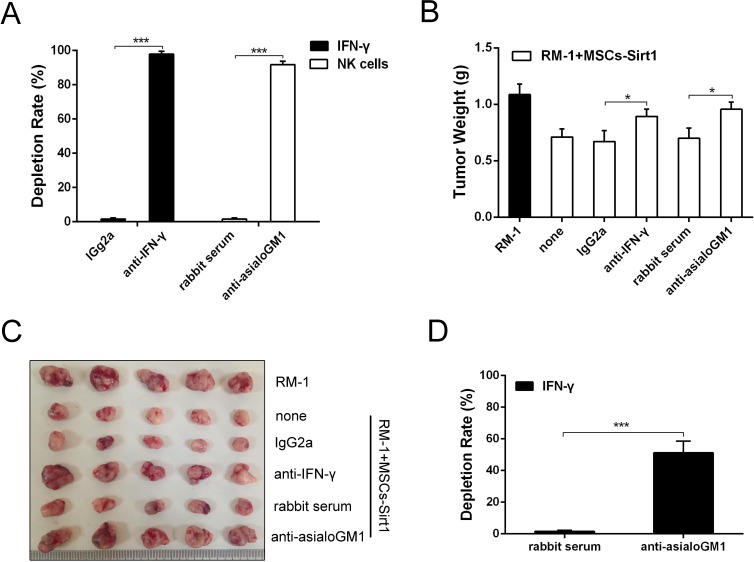
Effect of IFN-γ and NK cells on tumor inhibition **A.** Mice were depleted of IFN-γ and NK cells by anti-IFN-γ mAb and rabbit anti-asialoGM1 antiserum by intraperitoneal injection, respectively. The depletion rates were also texted through ELISA kit and flow cytometry. **B.** RM-1 tumor growth under IFN-γ depletion or NK cell subset depletion was examined again. Mice treated with an irrelevant monoclonal IgG2a or normal rabbit serum at the same dose were included as control. **C.** Representive tumors were presented. **D.** The depletion rate of IFN-γ was texted through ELISA kit after rabbit anti-asialoGM1 antiserum applied. Each group consists of 5 mice. *, *P* < 0.05; ***, *P* < 0.001.

To determine whether the MSCs-Sirt1-induced tumor inhibition depended on IFN-γ and NK cells, anti-IFN-γ mAb and anti-asialoGM1 antiserum were used to deplete IFN-γ and NK cells, respectively. As shown in Figure 3A, 97.9% depletion of IFN-γ and 91.8% depletion of NK cells were achieved. As shown in Figure 3B and 3C, neutralization of IFN-γ impaired the antitumor effect of MSCs-Sirt1, and the tumor growth was restored. Similarly, depletion of NK cells significantly weakened the antitumor effect, confirming that NK cells were critical to the MSCs-Sirt1-induced antitumor effect in tumor-bearing mice. To investigate the source of IFN-γ, we analyzed the influence of NK cells depletion on serum IFN-γ levels. We found that depletion of NK cells significantly decreased the serum IFN-γ level (Figure 3D). Together, these results suggest that MSCs-Sirt1 exhibit their antitumor effect via NK cells-secreted IFN-γ.

### MSCs-Sirt1 induce high levels of C-X-C motif chemokine ligand 10 *in vivo* and *in vitro*

**Figure 4:**
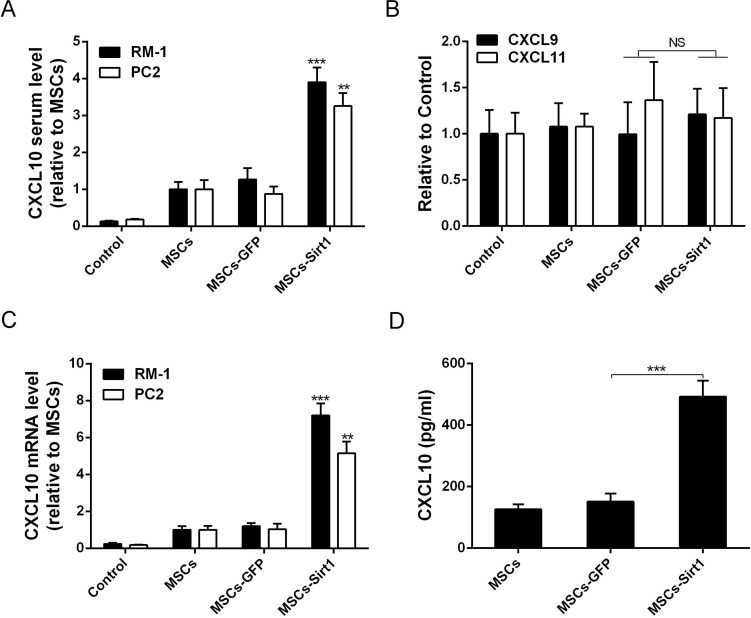
Evaluation of chemotactic factor CXCL10 production **A.** Serum samples of tumor-bearing mice were taken at the end of experiment. CXCL10 level was determined by ELISA kit and reported as ratio to MSCs group. **B.** Serum CXCL9 and CXCL11 levels in RM-1 tumor-bearing mice were reported as ratio to control. **C.** Real-time PCR was employed to examine CXCL10 expression level of tumors. Results were reported as ratio to MSCs group. **D.** ELISA assay was used to examine the expression of CXCL10 in conditioned medium of MSCs, MSCs-GFP and MSCs-Sirt1. The data presented are from three replicates as mean ± SD. **, *P* < 0.01; ***, *P* < 0.001; NS, *P* > 0.5.

The CXC motif chemokine receptor 3 (CXCR3), which is expressed on activated NK cells, regulates chemotaxis of NK cells, and induces their migration toward gradients of C-X-C motif chemokine ligand 9, 10 and 11 (CXCL9, CXCL10 and CXCL11)[17]. To analyze the mechanism by which MSCs-Sirt1 recruit NK cells for tumor inhibition, we measured the serum levels of chemokines CXCL9, CXCL10, and CXCL11. As shown in Figure 4A, serum CXCL10 level was significantly increased in MSCs-Sirt1 group compared with MSCs-GFP group. There was no significant difference in serum levels of CXCL9 and CXCL11 (Figure 4B). Expression of CXCL10 mRNA was also increased in tumor tissues of MSCs-Sirt1 group (Figure 4C). In addition, MSCs-Sirt1 cells *in vitro* released higher levels of CXCL10 compared to MSCs-GFP cells (Figure 4D). These results suggest that CXCL10 may be the key chemotactic factor recruiting NK cells for antitumor effect.

### MSCs-Sirt1 inhibit prostate tumor growth through CXCL10-recruited NK cells

**Figure 5:**
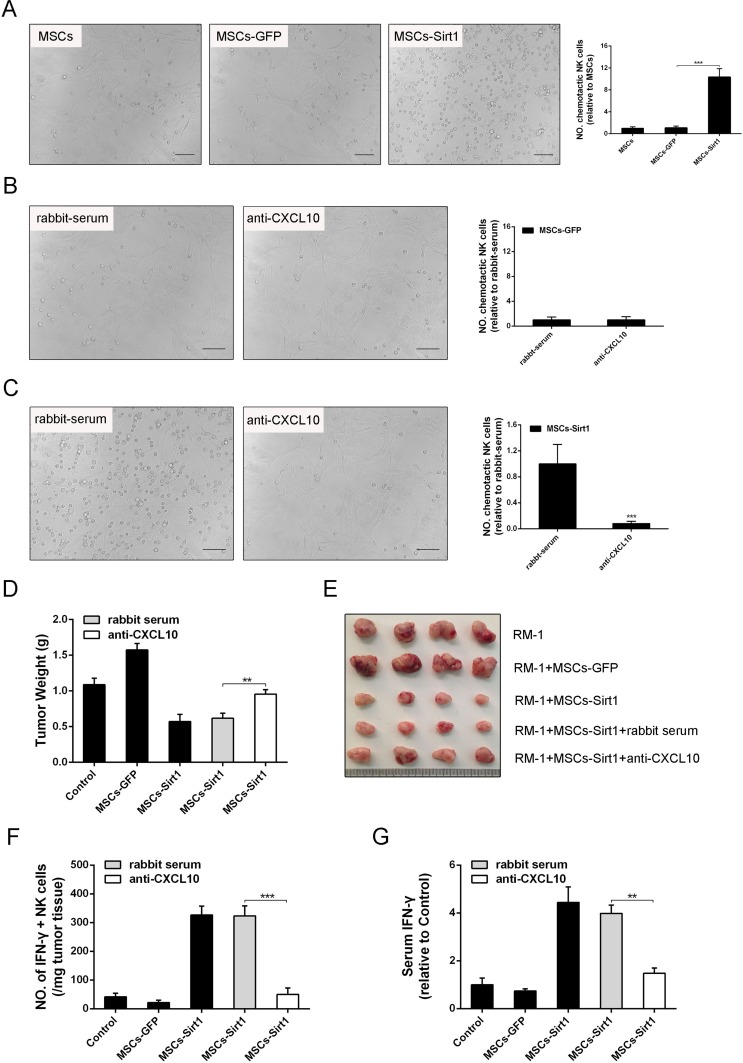
Effect of CXCL10 on RM-1 tumor suppression **A.** Conditioned medium of MSCs, MSCs-GFP and MSCs-Sirt1 were applied to detect their chemotactic effect on NK cells via transwell assay. NK cells were counted under a microscope (original magnification: ×200). The data obtained for plotting are from three replicates as mean ± SD. The effect of **B.** MSCs-GFP and **C.** MSCs-Sirt1 on NK cells migration was demonstrated through rabbit anti-murine CXCL10 (Abnova). Irrelevant normal rabbit serum at the same dose was applied as control. NK cell count was obtained as described above. **D.** RM-1 tumor weights were examined in mice injected intraperitoneally with rabbit anti-murine CXCL10 (2.5ug/5ul, Abnova) for CXCL10 depletion. **E.** Representive tumors were presented. **F.** Tumor infiltrating cells were isolated from RM-1 tumor tissues and then stained with antibody against NK cells, followed by intracelluar IFN-γ staining. **G.** Serum IFN-γ level was determined after NK cells depletion. Each group consists of 4 mice. **, *P* < 0.01; ***, *P* < 0.001.

To determine the CXCL10 chemotactic effect on NK cells, NK cells were co-cultured with MSCs-Sirt1 in a transwell *in vitro.* As shown in Figure 5A, MSCs-Sirt1 had a strong chemotactic effect on NK cells compared with MSCs-GFP. However, MSCs-Sirt1 treated with rabbit anti-murine CXCL10 antibody had attenuated chemotactic effect on NK cells (Figure 5B and 5C). These results indicate that MSCs-Sirt1 recruit NK cells through CXCL10 secretion.

In order to further elucidate the effect of CXCL10 on tumor inhibition, rabbit anti-murine CXCL10 antibody was used in the xenotransplant tumor model. IIFN-γ-secreting NK cells in tumor tissues, and serum IFN-γ levels. As shown in Figure 5F, depletion of CXCL10 dramatically decreased the number of tumor-infiltrating NK cells in tumor tissues of MSCs-Sirt1 treated mice. Similarly, the serum IFN-γ level was reduced in CXCL10-depleted MSCs-Sirt1 mice (Figure 5G). Together, these results indicate that CXCL10 recruits NK cells, thus contributing to the MSCs-Sirt1-induced prostate tumor suppression in mice.

### MSCs-Sirt1 induce macrophage activation in tumor tissues

**Figure 6:**
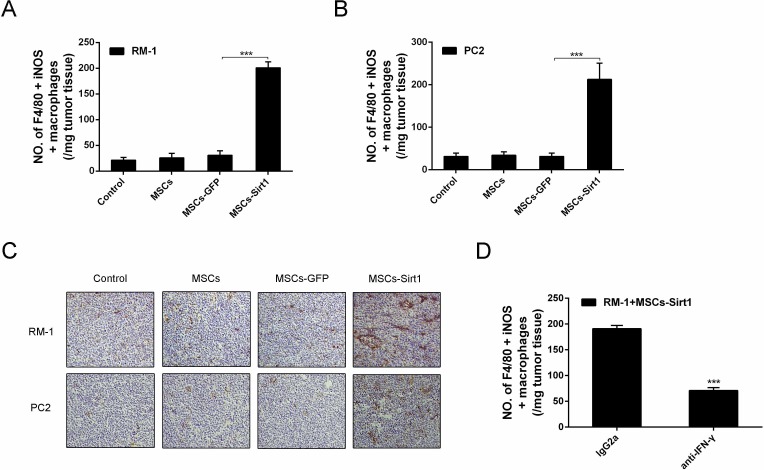
Macrophages activation in tumor regions Tumor infiltrating cells were isolated from **A.** RM-1 and **B.** PC2 tumors, then stained with anti-F4/80 antibody (Abcam), followed by intracelluar staining with anti-iNOS antibody. **C.** Immunohistochemistry against CD68 and iNOS in tumour tissues was observed by microscope (original magnification: ×200). **D.** Mice were intraperitoneally injected with anti-IFN-γ mAb or control lgG2a per 2 days until tumors excision. Tumor infiltrating cells were isolated and performed flow cytometric analysis. Each group consists of 6 mice. ***, *P* < 0.001.

IFN-γ activates macrophages and induces them to produce NO by iNOS, resulting in their increased tumoricidal activity [18]. Thus, we analyzed activated macrophages in tumor tissues by flow cytometry. As shown in Figures 6A, 6B and 6C, the number of activated macrophages was increased in RM-1 and PC2 tumor tissues of MSCs-Sirt1 group, compared to other groups. The infiltration was in response to IFN-γ production, since macrophage infiltration was greatly reduced when IFN-γ was neutralized in MSCs-Sirt1 treated mice (Figure 6D). These results indicate that the MSCs-Sirt1-induced tumor suppression is associated with the IFN-γ-induced recruitment and activation of tumoricidal macrophages.

## DISCUSSION

Mesenchymal stem cells (MSCs), also called multipotent mesenchymal stromal cells, exist in almost all tissues [5]. MSCs can migrate to tumor tissues [9] and create a tumor microenvironment promoting tumor development through escaping immune surveillance [10, 12, 16]. Since studies have shown that MSCs can migrate also to prostate cancer tissues and accelerate cancer growth and invasion [19, 20], the use of MSCs in PCa therapy remains limited. The goal of this study was to investigate the MSCs function in the regulation of PCa growth after overexpression of Sirt1. Interestingly, MSCs-Sirt1 suppressed RM-1 and PC2 prostate cancer growth *in vivo*, in contrast to the tumor promoting effect of MSCs. Our results showed that this antitumor effect was associated with the enhancement of immune inflammatory responses.

Reinforcing the function of the immune system can effectively control cancer progression. NK cells are an important component of innate immune system [21], and can be rapidly activated to attack tumor cells through producing inflammatory cytokines and chemotactic factors *in vivo* [22]. Lack of NK cells increases susceptibility to tumorigenesis [23]. In addition, several reports have demonstrated that tumor infiltration with NK cells leads to a good prognosis [24–26].

In this study, we have detected higher serum IFN-γ levels in MSCs-Sirt1 mice compared to other groups. We have also detected large numbers of IFN-γ-secreting NK cells in tumor tissues in the MSCs-Sirt1 group. Correspondingly, the antitumor effect of MSCs-Sirt1 can be greatly impaired after IFN-γ or NK cells depletion, suggesting that IFN-γ and NK cells are essential for the MSCs-Sirt1-induced tumor inhibition. Furthermore, we found that NK cells depletion could greatly decrease serum IFN-γ level in tumor-bearing mice. The data suggest that the antitumor effect of MSCs-Sirt1 may be mediated through JAK-STAT pathway, resulting in NK cells secreting IFN-γ [27]. The local release of IFN-γ recruits macrophages, and activates their tumoricidal activity [18, 28]. Consistent with the IFN-γ levels, we found an increase of activated macrophages in tumors in the MSCs-Sirt1 group.

CXCL10 is an inflammatory chemokine produced by different cell types in response to IFN-γ [29]. Our data demonstrated increased levels of CXCL10 in serum and tumor tissues of MSCs-Sirt1 mice. We also showed that MSCs-Sirt1 increased CXCL10 production, resulting in chemotaxis of NK cells *in vitro*. The CXCL10 secretion may be attributed to the activation of TLRs signaling pathways [30]. In addition, NK cells can produce CXCL10 when activated by IFN-γ [22, 31], thus promoting immune responses and antitumor effect [32]. Overall, our findings support the model that the increased production of IFN-γ and CXCL10 maintains MSCs-Sirt1-induced inflammatory responses and tumoricidal activation. However, the specific source of CXCL10 in tumor-bearing mice needs to be determined.

In view of the limited use of MSCs in cancer therapy, it is necessary to explore new strategies to expand the applications of MSCs. Our study demonstrates the antitumor effect of MSCs-Sirt1 on prostate cancer growth, and shows that the effect is mediated by NK cells and macrophages. These results indicate that MSCs-Sirt1 can be considered as a potential therapeutic choice for PCa.

## MATERIALS AND METHODS

### Cell culture

MSCs were derived from bone marrow flushed out of tibia and femur of 4-6 weeks old mice as described by Heng Zhu et al [33]. About 3×10^6^ cells per mouse were obtained after passage for three times. Cells were considered as purified MSCs and identified by adipocytes and osteoblasts differentiation as described in our previous studies [34–36]. MSCs were cultured in DMEM medium supplemented with 10% fetal bovine serum (FBS), 2 mM glutamine, 100 U/ml penicillin and 100 mg/ml streptomycin (all from Invitrogen, Carlsbad, CA) as previously reported [33]. Murine RM-1 and TRAMP-C2 (PC2) cells were cultured in RPMI-1640 medium (Invitrogen) with 10% FBS, supplemented with 100 U/ml penicillin and 100 mg/ml streptomycin. Murine NK cells were prepared by NK Cell Activation/Expansion Kit (Miltenyi Biotec) and cultured in RPMI-1640 medium supplemented with 10% FBS. All cells were cultured at 37°C in a 5% CO_2_ humidified atmosphere.

### Adenoviral vector construction

MSCs were infected with the adenoviral vector GFP-Sirt1 (Invitrogen) to increase the expression of Sirt1. As a control, MSCs were infected with GFP-Mock (Invitrogen). MSCs transfected with GFP-Mock (MSCs-GFP) or GFP-Sirt1 (MSCs-Sirt1) were harvested 48 hours after transfection for the next experiment.

### Animal studies

Male C57BL/6 mice, 6-8 weeks old, weighing 20-22 g, were obtained from the Shanghai Experimental Animal Center of the Chinese Academy of Sciences, Shanghai, China. Animal care was approved by the Institutional Animal Care Committee, and all procedures were performed in accordance with guidelines established by the Chinese Academy of Sciences' Committee on Animals. The mice were divided randomly into four groups to establish tumor-bearing model. Subcutaneous administration of the following cells was performed in the mice armpit area: Control group (1×10^6^ RM-1 or PC2 cells in 200 μl PBS), MSCs group (1×10^6^ RM-1 or PC2 cells and 2×10^5^ MSCs in 200 μl PBS), MSCs-GFP group (1×10^6^ RM-1 or PC2 cells and 2×10^5^ MSCs-GFP in 200 μl PBS), and MSCs-Sirt1 group (1×10^6^ RM-1 or PC2 cells and 2×10^5^ MSCs-Sirt1 in 200 μl PBS). All tumor-bearing mice survived until they were sacrificed on the 10^th^ or 18^th^ day at the end of experiment.

### Flow cytometric analysis

Tumor-infiltrating lymphocytes in tumor tissues were evaluated by flow cytometric analysis. At first, tumor tissues were cut into small pieces and the tissue fragments were incubated for 15 minutes at 37°C in HBSS solution containing collagenase type I (0.05 mg/ml), collagenase type IV (0.05 mg/ml), hyaluronidase (0.025 mg/ml), DNase I (0.01 mg/ml) and soybean trypsin inhibitor (1mg/ml) (Sigma). Cells were recovered by centrifugation and suspended in a fresh aliquot of the HBSS digestion solution for 15 minutes at 37°C. The cells were obtained through a 40 μm mesh sieve and recovered and washed with RPMI-1640 medium. They were further separated on a Ficoll-Paque gradient to remove dead cells; the left cells were used for cytometric analysis. Cells after stained for surface markers were fixed (BD Fix/Perm solution) and washed (BD Perm/Wash solution) according to the manufacturer protocol, and then were labeled with FITC-conjugated antibody for 30 min at room temperature in the dark. The stained cells were analyzed by a BD FACS Aria flow cytometer.

### Histopathology assessment

Tumor tissue sections were stained with Meyer's hematoxylin and eosin (H&E) and immunostained with primary antibodies including anti-CD49b (BioLegend) for NK cells, anti-CD68 (Abcam) for macrophages, and anti-iNOS (Abcam). Each sample was observed at a 200× magnification of microscopic field in 10 randomly selected areas.

### Depletion experiments

Anti-IFN-γ mAb (R4-6A2) was employed to deplete IFN-γ by intraperitoneal injection of 0.5 mg before subcutaneous administration and then 0.25 mg per 2 days. Rabbit anti-asialoGM1 antiserum (Wako Pure Chemical Industries) was employed to deplete NK cells with a dose of 20 μl in the same manner and schedule.

### NK activity assay

NK cytolytic activity was determined using mice splenocytes as effector cells, and YAC-1 cells as target cells at different effector/target (E/T) ratios. Target cells were labeled with ^51^Cr isotope (PerkinElmer). The percentage of specific lysis was calculated by the following formula: percent cytotoxicity = [(experimental release - spontaneous release by effector and target)/(maximal release - spontaneous release)] × 100.

### Real-time PCR

Real-time PCR was performed by mixing cDNA with primers and Maxima SYBR Green qPCR Master Mix (Applied Biosystems, Carlsbad, CA, USA) and using a Stratagene Mx3000P Real-time PCR System with supplied software (Applied Biosystems). The sequences of the primers were as follows: Sirt1 (forward: 5′-GCTGACGACTTCGACGACG-3′; reverse: 5′-TCGGTCAACAGGAGGTTGTCT -3′), CXCL10 (forward:5′-CCAAGTGCTGCCGTCATTTTC-3′; reverse: 5′-GGCTC GCAGGGATGATTTCAA-3′) and β-actin (forward: 5′-GGCTGTATTCCCCTCCA TCG-3′; reverse: 5′-CCAGTTGGTAACAATGCCAT GT-3′). β-actin was used as an internal control for RNA integrity and loading normalization.

### Enzyme linked immunosorbent assay (ELISA)

ELISA assays were performed using commercial ELISA kits (R&D Systems) according to manufacturer instructions. Assays were performed in duplicates, and readings were compared with standard curves obtained with standard protein provided with the kit. Means and standard deviations of concentrations in triplicate samples were compared by t-test.

### Transwell assay

The chemotactic effect of MSCs-Sirt1 on NK cells was assayed utilizing transwells with polycarbonate membranes (5 μm pore size, Cell Biolabs, San Diego, CA) in 24-well plates as described by Ren et al [37]. NK cells (1.5 ×10^5^) were added in the upper chamber containing 200 μL of serum-free medium, and 300 μL of MSCs-Sirt1 conditioned medium without serum was placed in the lower chamber. The plate was incubated at 37°C in a humidified atmosphere containing 5% CO_2_ for 3 hours. At the end of the experiment, the cells that passed through the filters were counted under a microscope.

### Statistical analysis

Final data were expressed as mean ± standard deviation (SD). Statistical analysis of the data was done by using GraphPad Prism 5. Student's t-test was used to compare between mean values of two groups. Value of at least *p* < 0.05 was considered statistically significant.

## SUPPLEMENTARY MATERIALS FIGURES


