# Supplementary material for: Mesenchymal stem cells overexpressing Sirt1 inhibit prostate cancer growth by recruiting natural killer cells and macrophages
Source: Oncotarget. 2016 Oct 18;7(44):71112–22. doi: 10.18632/oncotarget.12737 (PMC5342066; doi:10.18632/oncotarget.12737)
Supplement: Supplementary file 1 [file oncotarget-07-71112-s001.pdf]

Mesenchymal stem cells overexpressing Sirt1 inhibit prostate cancer growth by recruiting natural killer cells and macrophages

Supplementary Material

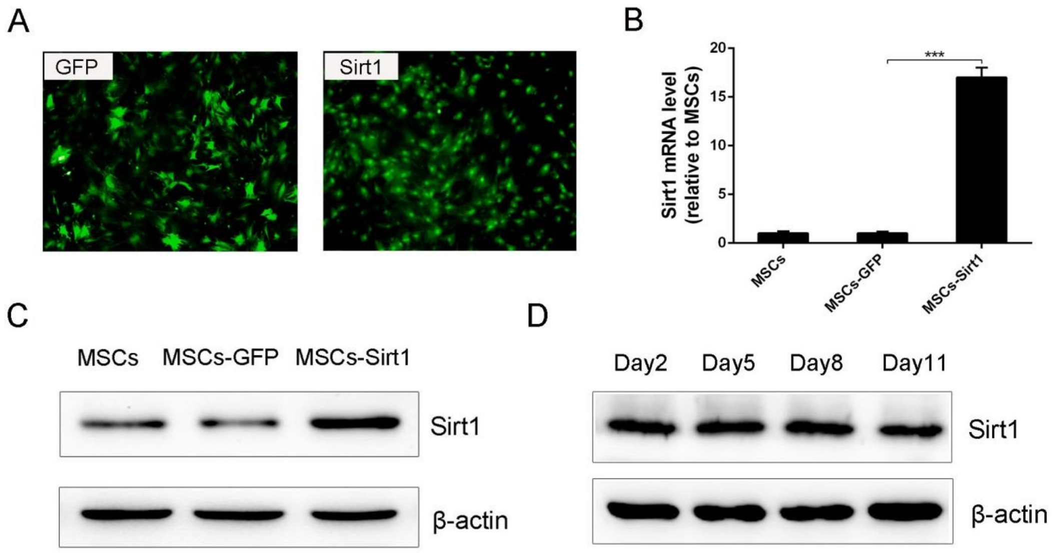

Supplementary Figure 1: Sirt1 expression level of MSCs infected with the adenoviral vector GFP-Sirt1. (A) MSCs infected with the adenoviral vector GFP-Sirt1 were detected by fluorescence microscope after infection 48h (original magnification: ×100). (B) Real-time PCR was employed to examine the expression level of Sirt1 after infection 48h and the data were reported as ratio to MSCs. (C) MSCs were lysed in RIPA lysis buffer (Beyotime) with 1 mM PMSF after GFP-Mock or GFP-Sirt1 infection 48h. The membranes were incubated with specific primary antibodies against Sirt1 or β-actin antibody (both from Abcam). Signals were visualized by chemiluminescent detection (Beyotime). (D) Changes about Sirt1 expression level of MSCs-Sirt1 with time going. ***, $P < 0.001$. MSCs-GFP, MSCs transfected GFP-Mock; MSCs-Sirt1, MSCs transfected GFP-Sirt1.

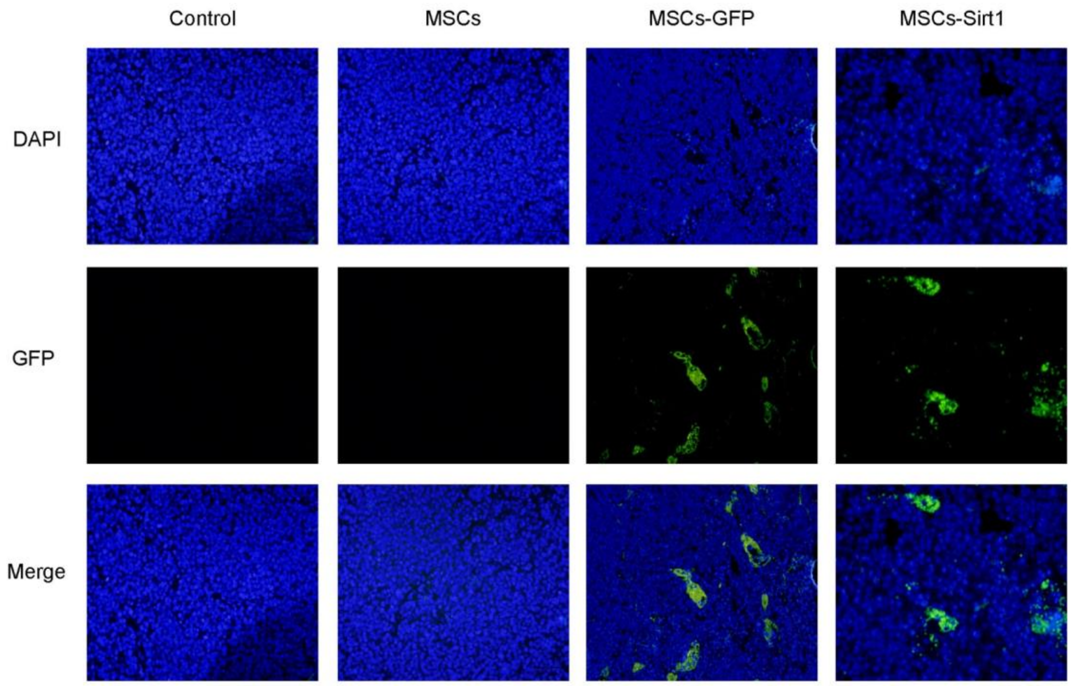

Supplementary Figure 2: MSCs with Sirt1 overexpression can survive the transplantation. RM-1 tumor tissues were sectioned and stained with primary antibodies anti-GFP (Abcam) for MSCs-GFP or MSCs-Sirt1. Sections were counterstained with DAPI (Beyotime) for nuclei staining. MSCs-GFP or MSCs-Sirt1 of tumor tissues were determined by fluorescence microscope assessment. Typical photographs were presented (original magnification: ×200).

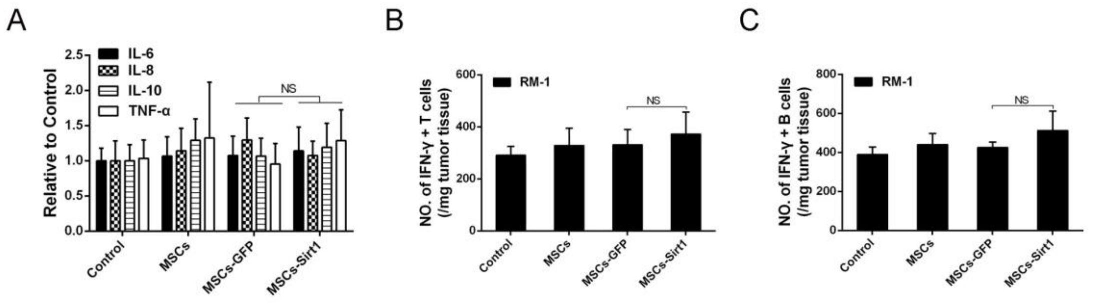

Supplementary Figure 3: Serum inflammatory cytokines levels and tumor infiltrating T and B cells evaluation. (A) Serum inflammatory cytokines levels were determined by Bio-Plex Pro^{TM} mouse cytokine assay kit (Bio-Rad Laboratories, USA) at 10th day since RM-1 cells subcutaneous injection. (B and C) Tumor infiltrating cells were isolated from RM-1 tumor tissues and were stained with anti-CD3 (Abcam) for T cells and anti-CD19 (BD Biosciences Pharmingen) for B cells, followed by intracelluar IFN-γ staining. Each group consists of 6 mice. NS, $P > 0.5$.
